# Three Ways That Non-associative Knowledge May Affect Associative Learning Processes

**DOI:** 10.3389/fpsyg.2016.02024

**Published:** 2016-12-27

**Authors:** Anna Thorwart, Evan J. Livesey

**Affiliations:** ^1^Department of Psychology, Philipps-Universität MarburgMarburg, Germany; ^2^School of Psychology, The University of Sydney, SydneyNSW, Australia

**Keywords:** associative learning, causal learning, expectation, prediction error, blocking

## Abstract

Associative learning theories offer one account of the way animals and humans assess the relationship between events and adapt their behavior according to resulting expectations. They assume knowledge about event relations is represented in associative networks, which consist of mental representations of cues and outcomes and the associative links that connect them. However, in human causal and contingency learning, many researchers have found that variance in standard learning effects is controlled by “non-associative” factors that are not easily captured by associative models. This has given rise to accounts of learning based on higher-order cognitive processes, some of which reject altogether the notion that humans learn in the manner described by associative networks. Despite the renewed focus on this debate in recent years, few efforts have been made to consider how the operations of associative networks and other cognitive operations could potentially interact in the course of learning. This paper thus explores possible ways in which non-associative knowledge may affect associative learning processes: (1) via changes to stimulus representations, (2) via changes to the translation of the associative expectation into behavior (3) via a shared source of expectation of the outcome that is sensitive to both the strength of associative retrieval and evaluation from non-associative influences.

## Introduction

Associative theories of learning offer a powerful account of the way animals and humans assess the relationship between events and generate expectations about the future. They assume that we reflect our knowledge about the predictive relationships between events in associative networks, which consist of mental representations of these events and the associations that link them. These events could be predictive cues and subsequent outcomes in the case of Pavlovian learning or actions associated with antecedents and consequences in the case of instrumental learning. Through observing the co-occurrence of cues and outcomes, an individual learns the associations between them in such a way that the presence of a predictive cue brings to mind the outcome and thus informs subsequent behavior by generating an outcome expectation.

Associative accounts have been applied to many widely replicated learning phenomena. The focus of theory development over the last 50 years has included explaining how simultaneously presented cues might compete for association, how selective attention affects and is affected by learning, and explaining how association formation could be a simple but effective means of tracking statistical contingencies between events rather than merely tracking their temporal coincidence. Not surprisingly, many associative learning models provide detailed and compelling explanations for these phenomena. Central to many of these explanations is the notion of *prediction error*, the discrepancy between what the associative system predicts will happen next and what is then actually experienced. The prediction error thus captures an experienced violation of expectations and we return to this concept and its widespread use in associative learning theory later. (Note also, we will use the terms expectation and prediction interchangeably). The term expectancy will usually refer to the explicit judgement of outcome expectations.

Despite the relative success that associative learning models have enjoyed in both explaining and predicting phenomena observed in conditioning and contingency learning studies, there are clearly also many influences on learning that associative models simply do not capture. In this article, we review some of these influences and speculate on how they might influence the operations of an associative learning system, assuming that such a system forms a core part of human learning and cognition more generally. A number of theoretical and empirical papers published in the last decade have approached this question and reviewed relevant literature in causal and contingency learning (e.g., Shanks, 2007; Mitchell et al., 2009; Boddez et al., 2014). The current paper does not intend to systematically and comprehensively review the same body of literature or provide a critique of the theoretical views proposed by these authors. Instead, our focus will be on outlining and discussing ways non-associative knowledge might influence the operations of an associative learning system without changing its fundamental principles. In doing so, we hope to provide a means of evaluating the contribution of theories based on associative networks to explaining complex behavior more broadly. We would argue that this is particularly relevant to human associative learning, where influences on behavior are clearly more complex than formal associative models can explain in isolation but where there is still support for the existence of association formation mechanisms. Some of the traditional sources of evidence have failed to convince all theorists that it is necessary to posit association formation as being mechanistically distinct from inferential reasoning or higher order cognition in general. For instance, the notion that associative learning can occur in the absence of awareness is still as contentious as ever (see Goujon et al., 2015; Colagiuri and Livesey, 2016; Vadillo et al., 2016 for a recent iteration of this debate concerning implicit learning in visual search). Nevertheless, a number of results (e.g., Morís et al., 2014; Perruchet, 2015; Cobos et al., 2016) suggest that associative learning mechanisms are separable from other cognitive sources of expectation in at least some circumstances and could represent the operation of an independent system. This possibility is certainly plausible enough to warrant a more in-depth consideration of how associative and non-associative sources of prediction might interact.

## Expectancy and Judgment in Human Causal Learning

Studies of causal and contingency judgements are concerned with the way humans make explicit assessments of the predictive and causal relationships between events. These events may consist of a particular outcome in a fictitious scenario, such as an allergic reaction suffered by a patient, and the cues that may cause or predict that outcome, e.g., foods eaten by the patient. Participants will receive on a trial-by-trial basis information about what the patient has eaten. They are then requested to make a choice between different possible allergic responses that the patient might experience, e.g., “no allergic response,” “rash,” and “fever,” to indicate their expectation, and receive feedback whether their expectation was correct and which symptoms the patient actually suffered after eating these foods. In a test phase after several trials of training, participants might be additionally asked to rate the relationship of certain foods with a certain allergic symptom on a scale from “not predictive/causal” to “highly predictive/causal.”

One line of research has been to relate this kind of causal learning to classical conditioning and by this to associative accounts of learning. In addition to the apparent parallels in both the procedure and the content of learning – participants learn to predict future events based on their relationships with preceding events – many behavioral effects can be observed in both classical conditioning and human causal learning paradigms. However, when researchers started to investigate factors controlling learning in these kinds of procedures, critical factors quickly emerged that were not easily captured by associative models of learning, factors relating to “non-associative” knowledge relevant to the learning situation (see De Houwer, 2009).

## Associative and Non-Associative Knowledge: A Working Definition

For the purpose of this paper, we will define *associative* knowledge as knowledge that can be derived merely from the statistical relationships among the relevant cues and outcomes. All knowledge that goes beyond this is then seen as *non-associative*. This concerns both the way this information is obtained and the content of the information. Non-associative knowledge includes information given verbally (i.e., by instruction, other people’s accounts, prior semantic knowledge), relational information inferred from the co-occurrence of other, separately presented cues and outcomes, (e.g., whether other outcomes are predicted reliably by other cues), or information implied by other aspects of the experimental procedure (e.g., spatial position of cues and outcome on the screen, the format of the test question). We also regard information about properties of the associative links other than the statistical contingency, like their causal nature or the additivity of their effects, as non-associative, as well as information about properties of the cues and outcomes (e.g., whether the outcome is binary vs. continuous, and if continuous, whether it is observed at a maximal or submaximal intensity). These and many other factors that are related to the individual’s understanding of, or engagement with, a given context may impact on the learning of causal relationships but are rarely captured satisfactorily by formally quantified associative learning mechanisms. These factors all involve prior knowledge of one form or another, and it must be assumed that their influence thus depends very heavily on prior learning. The point of defining them as non-associative is not to make particular claims about their content or the mechanism by which they were initially acquired, but rather to acknowledge that their often substantial impact on new learning is not formally captured in existing models of association formation^1^.

## Blocking in Human Causal Learning and Its Associative Explanation

In the following, we rely heavily on research on the *blocking effect* (Kamin, 1968). In terms of its influence on the development of new theories, Kamin’s blocking effect in Pavlovian conditioning is historically one of the most important phenomena. The blocking effect is also an often cited example for a basic learning effect that is regularly reported in human causal learning. It has been one of the empirical cornerstones for the argument that the same underlying learning processes are controlling learning in both conditioning and in human causal learning (Dickinson et al., 1984) and it is therefore not surprising that a particularly high number of studies have investigated non-associative influences on human learning in the context of blocking paradigms.

In a simple blocking experiment (see also **Table 1**), participants might first observe that cue A results in the occurrence of the outcome (A+). In a subsequent phase they may also observe that cues A and B together result in the occurrence of the outcome (AB+). On other trials, they observe that cues C and D together also result in the occurrence of the outcome (CD+). When asked to judge whether B causes the outcome, participants will often give a rating that is substantially lower than their rating for either C or D, even though all three cues have resulted in the outcome on an equal number of occasions. For example, if participants first experience that apples cause an allergic reaction in a Patient X and afterward that two different food combinations, one comprising apples (A) and beans (B) and one carrots (C) and dates (D), will both lead to an allergic reaction, they will rate beans as less likely to cause the allergic reaction on its own than carrots or dates (i.e., B < C or D, see for example, Luque et al., 2013).

**Table 1:** A typical set of contingencies displayed to participants as part of a blocking experiment.

	Stage 1	Stage 2	Test
Blocking cues	A+	AB+	B?
Control cues		CD+	C? D?


*Letters A–D refer to predictive cues, + refers to the presentation of the outcome, ? to the absence of feedback on test.*

**FIGURE 1:**
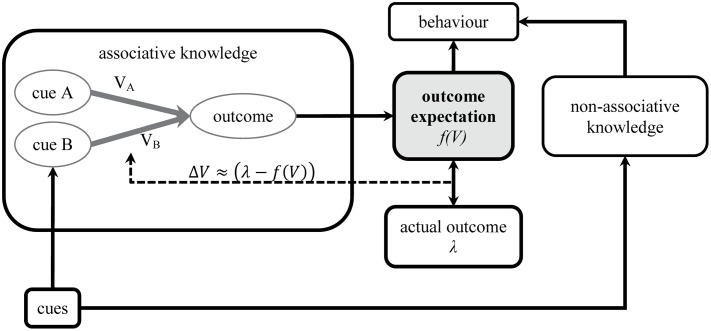
**Simple associative network and its interaction with the events in the world**.

**Figure 1** depicts an example of a simple standard associative network after training and its interaction with the events in the world. The network inside the box on the left side consists of two cues, A and B, which are each connected via associations to the representation of the outcome. As a result, the presence of a predictive cue, represented by the black rectangles outside the box, activates not only its own representation within the associative network but brings also to mind the associatively connected representation of the outcome. Associative models claim that the strength of this associative retrieval of the outcome is a key source of evidence in making a prediction about the outcome. The outcome is rated as likely to occur after certain cues because these cues activate its representation and thus result in an expectation of the outcome. As the strength of this associative retrieval, and thereby the outcome expectation, is a function of the strength of the associative links between the presented cues and the outcome, *f(V)*, differences in responding are based on differences in associative strength.

Most associative accounts furthermore rely on the *prediction error* in some way to establish the associations. Broadly speaking, changes in associative strength, ΔV, are proportional to the error made in the prediction of the outcome, i.e., the violation of the expectation. Every time a prediction is made, it is compared to the actual outcome, represented via the value assigned to λ (e.g., λ = 1 if the outcome is present; λ = 0 if absent). The resulting prediction error, that is, the difference between the actual outcome and the outcome expectation, is given by the generalized error term [λ - f(V)], and is used to optimize the associative links such that the error is minimized in future predictions. Most models therefore agree that the current prediction plays a key role for the formation and further adjustment of the associative links. Different models assume different ways of combining the associative effects of several cues, that is, when several cues are presented together at the same time, for example A and B in an AB compound. The Rescorla and Wagner (1972) model and most others like it rely on an additivity assumption, that is they assume that the associative effect of cue A and cue B will be the same when they are subsequently presented within the compound AB and will simply sum together, *f*(V) = Σ*V*. The equation controlling the changes in associative strength can thus be expressed in the following way: ΔV ∼ (λ - ΣV).

According to associative theories of blocking, participants will develop a strong association between the cue A (apples) and the outcome (allergic reaction) in the first stage of a blocking experiment, so that, whenever A is presented, the outcome will be retrieved and correctly expected. If the outcome is already expected and subsequently occurs, then minimal learning will take place because the outcome was not surprising (i.e., no prediction error). The same is true for the AB compounds in the second stage. Cue A will again strongly activate the outcome representation and result in a strong expectation of the outcome. Therefore, the prediction error in AB+ trials is already minimal and further correction of the associative links of either A or B with the outcome are soon unnecessary. Learning about B will be blocked by the previous learning about A, and B will not develop a strong association with the outcome. In contrast, the control cues C and D will not retrieve the outcome representation at the beginning of the second stage of the blocking procedure and will therefore not generate a strong expectation of the outcome in CD+ trials. The resulting prediction error will in turn fuel the formation of associative links between C, D and the outcome. At the end of the experiment, C and D will each result in a stronger associative retrieval of the outcome than B (because *V*_B_ < *V*_C_/*V*_D_) and thus in a stronger prediction, even though they were all paired the same number of times with the outcome. The concept of *prediction error* as a determinant of learning is thus closely linked to the blocking effect and furthermore, prediction error has been shown to be important if not indeed causal for blocking (Steinberg et al., 2013). Blocking is regarded as an instance of cue competition because A and B (and likewise C and D) arguably compete over the association with the outcome. B loses this competition as it is paired with A, which had a head start by virtue of its prior individual pairing with the outcome.

## Some Known Effects of Non-Associative Knowledge

From the description of a simple associative network, it should be apparent that participants that receive the same cue-outcome pairings should show the same learning as this is the only kind of information on which the formation of associations and thus the expectation is based. As already pointed out, however, it is well established that non-associative knowledge affects learning and decision making. One classic demonstration of the effect of verbal information on causal learning was provided by Waldmann and Holyoak (1992). Their experiments were designed to create two learning tasks that were equivalent at the associative level – that is, identical in terms of the statistics of the events involved – but differed in terms of the general causal information conveyed in the cover story. All participants in Waldmann and Holyoak’s study received the same cue-outcome combinations during Stages 1 and 2 of a blocking experiment. However, the cover story established either a predictive or diagnostic learning situation for these cue-outcome pairings. While the cues were always the same, participants in their predictive task had to learn which cues would *cause* a new kind of emotional response in observers. In contrast, participants in the diagnostic task saw the same cues but redefined as symptoms of a disease and had to learn which symptoms *were caused* by the disease. Even though subjects saw identical cues and cue-outcome pairings, they rated the critical target cue differently in the diagnostic and in the predictive condition. Specifically, participants given the diagnostic scenario gave the target cue, B, a stronger rating. As Waldman and Holyoak’s study did not include the appropriate control cues for blocking, C and D, drawing a conclusion on the blocking effect is not possible. However, similar subsequent experiments have replicated the effect of causal model, implemented through instructions and prior knowledge, on blocking and other effects (e.g., Waldmann, 2000, 2001; Luque et al., 2008; Blanco et al., 2014; but see Shanks and Lopez, 1996; Thorwart and Lachnit, 2010).

Another line of experiments has demonstrated an effect of inferential reasoning on blocking. These experiments show how information about the causal relationship between cues and outcomes influences learning. In De Houwer et al. (2002) experiments, participants had to rate how likely it was that a tank would be destroyed (i.e., the outcome) if a certain weapon was fired (i.e., a causal cue) or if an indicator lit up (i.e., a predictive cue). Weapons and indicators were represented by the same abstract visual cues that were present during training shortly before the possible destruction. Nevertheless, participants rated the relationship between them and the destruction of a tank differently. Beckers et al. (2005b) replicated this result in 4-year-old and 8-year-old children with a scenario about predicting rain, rather than exploding tanks. A related series of studies addressed how assumptions about the additivity of the causal effects of cues may determine the strength of the blocking effect (Lovibond et al., 2003; Beckers et al., 2005a). An additivity rule would state that, if two cues cause the outcome separately, the outcome should be even stronger when both cues are present at the same time. This assumption permits the application of simple deduction such that on observing that A and B together do not cause a more severe allergy than A alone, B must therefore not cause the outcome. Consequently one would expect to observe blocking from inferential reasoning alone if participants hold this assumption. A non-additivity rule would instead reflect the belief that adding a second cue does not increase the likelihood or strength of the outcome if this is already predicted by the first cue, even if both cues are predictive on their own. Pretraining and verbal instructions were successfully employed to shift participants’ beliefs in one or the other direction and these experiments showed repeatedly that affirming the additivity rule strengthens the blocking effect.

Models of associative learning are designed to account for blocking but, as these examples show, the presence of the effect itself varies considerably across procedures, and in ways that seem to be more consistent with cognitive processes that differ considerably from the simple principle that learning is proportional to prediction error. For the theoretical approach typified by associative networks, the challenge posed by these results is not the fact that they show other cognitive factors play a role in controlling behavior. For instance, nowhere have associative models explicitly assumed that other mental processes cannot produce cue competition effects (symbolized by the arrow from cues to non-associative knowledge in **Figure 1**). But the fact that associative models do not speak to these non-associative factors works against their relative utility as accounts of human learning, since there are clearly important properties of human learning, judgment, and behavior that they fail to capture. Clear evidence exists that non-associative factors influence associative learning in the laboratory. It *might* be the case that this evidence reflects a thin veneer of cognitive penetrability on an otherwise highly regular and lawful set of learning principles that capture real-life learning quite well. After all, knowledge that one is participating in a psychology experiment must surely encourage introspection and careful thought. Alternatively, and more worryingly for the conventional associative approach, this evidence may be symptomatic of broad, general and far-reaching sensitivity to a host of factors that are poorly accounted for by associative learning networks. Therefore, even if one is to retain the association-formation approach in theorizing about human learning, there is a need to better understand how other factors play a role in human learning.

## How Might Non-Associative Knowledge Influence An Associative Network?

Since non-associative knowledge can clearly influence associative learning phenomena, including those that form the basis of contemporary prediction-error models, it is tempting to discard the notion that we possess a system dedicated to mental association in the manner described by associative networks. Indeed some authors have already reached this conclusion (Mitchell et al., 2009). They too assume that the expectation of the outcome will inform our behavior, but this expectation is based on generating and evaluating propositions in deductive reasoning processes. However, an alternative approach, and one that we think is still instructive, is to ask how non-associative knowledge could impact upon learning, expectations, and behavior if we assumed that a general-purpose associative system was still in place. How could the non-associative knowledge influence the operations of such a system and what would be the implications if it did so? Here we consider briefly several possible ways in which this could occur.

### Non-associative Knowledge May Change the Inputs to an Associative Network

One might account for the variations in the blocking effect in causal learning by suggesting that the formation of associations is sensitive to parametric differences. Certainly, most associative learning models generate parameter-specific predictions about various learning phenomena, meaning that quantitative parameter variations can produce different effects without fundamentally changing the inner workings of the network itself. They affect what is happening but not how it is happening. Often the manner in which stimuli are represented within an associative network can be critical for how learning takes place. For instance, although associative learning models generally predict blocking, the strength of the predicted effect can vary widely according to assumptions about how the stimuli are mentally represented and how quickly learning occurs during training. These assumptions are captured in parameters like the associability of the cues. If non-associative knowledge alters such parameter values, it would affect learning without replacing or even fundamentally changing the learning mechanism that is assumed to have worked and survived successfully throughout evolution. However, why and how should non-associative knowledge influence quantitative parameters of the network?

**FIGURE 2:**
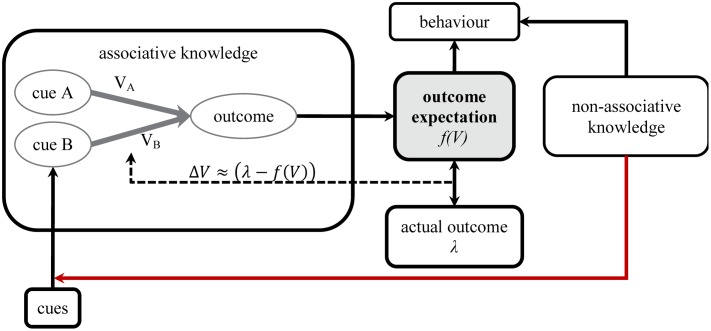
**Non-associative knowledge may change the inputs to an associative network (indicated by connection in red)**.

Associative models generally assume that physical properties of the cues and the outcome influence parameters like their associability such that there is a link between basic perceptual principles and the determinants of learning (Annau and Kamin, 1961; Mackintosh, 1976; Redhead and Pearce, 1995). Many theorists take a further logical step by assuming that basic cognitive operations like attention also determine key aspects of stimulus representation in the learning system. That is, the mental representations that engage in learning reflect information subjected to limited sensory processing, which is selectively biased by attention. Theorists have often assumed that selective attention affects learning in other animals just as it appears to in humans (e.g., Lashley and Wade, 1946; Sutherland and Mackintosh, 1971; Mackintosh, 1975; Pearce and Hall, 1980). Therefore, to the extent that beliefs derived from non-associative knowledge can affect attention and perception, those beliefs may also impact upon learning within an associative network, even if the operations of that network are relatively automatic (**Figure 2**).

Work on the learned predictiveness effect clearly demonstrates an effect of instructed attention on selective learning (Mitchell et al., 2012; Don and Livesey, 2015; Shone et al., 2015). The learned predictiveness effect is a widely observed learning bias toward previously predictive cues in novel situations (see Le Pelley et al., 2016 for a recent review). The effect is generally attributed to an attentional shift that occurs as a natural consequence of acquiring associative knowledge about those cues. As cues are associated with outcomes, attention to more predictive cues is enhanced, resulting in faster learning for those cues within the associative network. However, Mitchell et al. (2012) demonstrated that explicit instructions manipulating participants’ beliefs about the predictiveness of cues in a second learning phase had significant effects on their learning of cue–outcome contingencies. After learning about the predictiveness of the stimuli in a trial-and-error fashion (which we assume led to acquisition of associative knowledge), participants in a “continuity” group received instructions at the start of Phase 2 that the cues that were predictive in Phase 1 were also likely to be predictive in Phase 2. A “change” group received opposing instructions, that the cues that were predictive in Phase 1 were unlikely to be predictive in Phase 2. Interestingly, participants in the “change” group showed a strong reversal of the learned predictiveness effect. That is, more was learned about previously non-predictive cues than previously predictive cues in Phase 2. Subsequent studies have partially replicated this sensitivity to instructions, though have typically found much weaker instructed reversal effects accompanied by a continued influence of biases established in Phase 1, despite clear evidence that the participants have read and understood the instructions (Don and Livesey, 2015; Shone et al., 2015). While Mitchell et al. (2012) favored an explanation purely based on conscious reasoning processes, where participants deliberately attend to the cues they believe are important, a viable alternative is that attentional processes are brought under conscious control and thus let non-associative knowledge influence the course of subsequent learning. This source of influence does not necessitate that non-associative expectations fundamentally change the operations of the associative network itself, merely what it receives (Livesey and Harris, 2009). In other words, a cue that possesses relevance merely because the instructions have enhanced its importance may be better or more fully represented in an associative network (i.e., have greater salience) because the individual is deliberately attending to it.

This might also go some way to explain some instances where the blocking effect appears to be unreliable or completely absent. In addition to the associative processes explained above, some theories assume that blocking is partly governed by a lack of selective attention to the blocked cue, either because it is redundant (Mackintosh, 1975) or because the outcome is predictable (Pearce and Hall, 1980). If non-associative factors influence selective attention, they may provide a means by which attention to the blocked cue is enhanced (or reduced even further), which could alter the likelihood of observing a blocking effect considerably even if learning were still primarily based on association formation.

In addition, if non-associative knowledge can affect the way stimuli are represented then this knowledge may also change the manner in which associative retrieval generalizes from A to AB at the beginning of Stage 2 learning and from the compounds to the single stimuli presented on test (Livesey and Boakes, 2004; Thorwart and Lachnit, 2009, 2010). Several authors have suggested that pretraining, task instructions, and spatial stimulus characteristics can alter the encoding strategy that participants use or the way they mentally represent cues, which in turn affects generalization between compounds and individual cues (e.g., see Melchers et al., 2008 for a review). The potential for these changes in stimulus representation to impact on learning is sometimes discussed in terms of flexible shifting between elemental and configural learning (Melchers et al., 2008) or shifts within an elemental learning system (e.g., Wagner and Brandon, 2001; Livesey and Harris, 2008; Thorwart et al., 2012, 2016). Such changes in stimulus representation reduce generalization from A to AB and thus result in a weak expectation of the outcome in AB+ trials. The resulting increased prediction error supports considerable further correction of the associative links of both A and B with the outcome. This change in stimulus encoding would also affect the generalization from trained compounds AB+ and CD+ to individual test cues B, C, and D, which may result in overall weak expectation and a smaller blocking effect in test, where blocking is generally measured by the difference between the rating of B and the mean rating of C and D. If all ratings are low due to reduced generalization from the training compounds AB and CD, the blocking effect will be small as well. No matter how the global properties of stimulus representation operate, the broader issue at hand is that generalization between different trial types might vary according to various sources of non-associative knowledge that affect stimulus encoding, which in turn impact on the expectation of the outcome when a new but related trial type (e.g., AB+) is experienced.

Finally, how and what information is sampled by the learner affects learning (Matute, 1996; see Fiedler and Juslin, 2006, for similar arguments in relation to decision making) and it is known that sampling strategies can be modified through verbal instructions (Matute, 1996; Blanco et al., 2012) or the amount of personal involvement (Yarritu et al., 2014). This influence is clearest in instrumental tasks where the learner’s actions directly control the delivery of outcomes and thus also the opportunities to observe relationships between action and outcome. For instance, in contingency judgment experiments where participants are asked to judge the degree of control of an action over the occurrence of an outcome, participants often perform the action relatively frequently (e.g., on considerably more than 50% of trials), which in turn limits the opportunity to learn about the likelihood of the outcome in the absence of the action and creates circumstances that favor overestimation of the association between the action and outcome. Changes in action strategy can thus directly influence the quality of the evidence for statistical relationships between events, and these strategic changes could be initiated by any number of non-associative manipulations.

### Non-associative Knowledge May Change How Associative Outputs Translate to Beliefs and Behavior

The clearest evidence that associative and non-associative knowledge might provide dissociable expectations at a behavioral level comes from studies that compare explicit predictions and ratings with other behavioral measures such as response priming and conditioned responding that gauge expectation less directly. One example is the Perruchet (1985, 2015) effect, where within the same experiment and indeed the same trial, diverging response patterns can be obtained in two behavioral systems (for example eye blink conditioning and causal rating; for details see below). Cobos et al. (2016) observed diverging “associative” and “non-associative” response generalization in cued response times and verbal ratings, respectively and Morís et al. (2014, Exp 4) found that non-associative knowledge, given by instruction, affects verbal judgements but not responses in a recognition priming-based test. But a related and in many ways more difficult question is how associative predictions might generate explicit judgements.

Most associative models generate predictions about behavior based on the summed associative strength of the cues that are active, or the activation of the representation of the outcome itself, outputs that we will refer to as *associative predictions*. Because they are usually intended to apply to a wide range of behavioral paradigms, few associative models provide formal rules for translating these associative predictions into specific behaviors. Fewer still provide precise rules for how associative predictions should be translated into judgements or verbal behaviors of the variety that can only be meaningfully measured in human learning. As such, when model predictions are tested empirically, they are usually expressed as ordinal hypotheses rather than precisely quantified predictions.

A problem thus still remains in characterizing how associative predictions are conveyed in the explicit expectations of the individual and whether the relationship between the two should be expected to be consistent across different experimental situations. It might well be expected that simple memory and retrieval mechanisms determine our judgements in at least some situations. The classical associationist view is that a cue might be judged as being the cause of an outcome to the extent that the presence of the cue brings to mind the idea of the outcome. Similarly we might expect a particular outcome to occur simply because a representation of that outcome has been activated via its associations with other cues that are present at the time.

**FIGURE 3:**
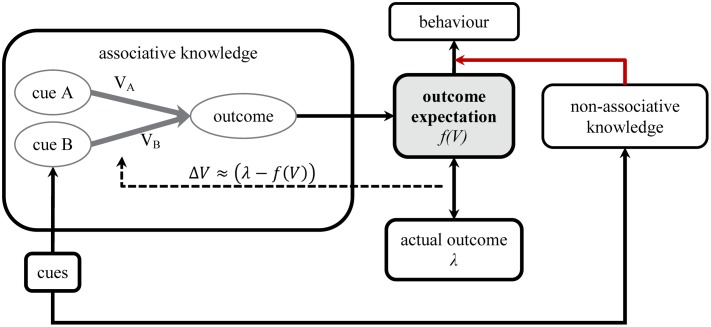
**Non-associative knowledge may change how associative outputs translate to beliefs and behavior (indicated by connection in red)**.

Theoretically, this relationship between associative retrieval and causal rating could be regarded as an immutable property of a system that integrates memory with an understanding of causal structure. Alternatively, it may be that the fluency of memory retrieval serves as just one source of evidence on which judgements about causation and expectations about future events are based, as conclusions based on non-associative knowledge serves as a second (**Figure 3**). In some circumstances, associative activation of the outcome may form the strongest available evidence about what is going to happen when a cue is presented, or the strongest indicator of how the individual should behave. But under other circumstances, for instance where it is very clear that a deductive reasoning process should be used, associative memory retrieval may play a relatively minor role. Thus the relative strength of non-associative knowledge may play an important role in how associative predictions translate to overt judgements and predictions. One might then assume that associative learning in the form characterized by associative networks exists and operates fairly consistently across different individuals and contexts, and that most of the variance in causal judgments results from non-associative factors having an influence on performance, for instance in the interpretation of associative memories and their translation into explicit behavior.

This possibility again does not imply that the internal workings of the associative network need be materially affected by expectations derived from non-associative knowledge. It merely assumes that associative predictions do not always have a strong influence on behavior. Returning to the blocking example, it is possible that the observed sensitivity of predictive ratings to non-associative information about causality (e.g., blocking is more readily observed in causal scenarios than non-causal scenarios) means that associative retrieval plays no part in determining the judgements made in either scenario. But it could also mean that associative retrieval plays a greater a role under some instructional and task conditions than others (e.g., Sternberg and McClelland, 2012). For instance, perhaps judgments that feel more naturally intuitive or familiar to the individual allow a greater influence of associative predictions, particularly among individuals who are disposed to making intuitive judgments already (Livesey et al., 2013). Support for such an influence of non-associative knowledge may be found in studies by Matute et al. (1996), Vadillo et al. (2005) and Vadillo and Matute (2007), which showed that the precise wording of the test question does have an influence on judgements. For example, Matute et al. (1996) found that the relative-validity effect, another cue competition effect related to blocking, appears when subjects are asked to rate whether the target cue X is a cause or an indicator of the outcome, but vanishes when participants are asked to rate to what extent cue X and the outcome co-occurred. Similarly, Gredebäck et al. (2000) found a significant cue competition effect when participants were asked about the predictive value of the cue, as well as when they were asked about the causal relationship between the cue and the outcome. However, the cue competition effect did not reach statistical significance when participants were asked about the probability of the outcome given the cue, nor when they were asked about the frequency of cue–outcome pairings.

Many of the results that we have discussed thus far, including those that show a sensitivity of blocking to causal model, contain single dissociations in which the behavioral ratings in one condition are generally closer to ceiling (e.g., Waldmann, 2001) and therefore change the likelihood of observing differences between ratings for reasons that might be to do with the measurement scale rather than the underlying process. For example, ratings in non-causal scenarios tend not to show blocking effects as readily as causal scenarios, specifically because the rating for the blocked cue is higher. If there were differences in how participants regard the blocked cue and the control cues that were in fact equivalent under causal and non-causal scenarios, it is reasonable to assume that those differences would appear to be weaker, possibly even non-existent, if ratings were generally near ceiling anyway. Thus an observation that blocking is weaker in non-causal scenarios could be achieved simply by assuming that participants use the scale differently in the two scenarios, without making any assumptions about changes in underlying process. Although we do not necessarily favor an explanation purely in these terms, it is worth pointing out that the evidence suggesting sensitivity to non-associative influences on causal learning is often consistent with multiple explanations, and at least some of these explanations do not assume that anything fundamentally different is happening in terms of learning and memory when non-associative knowledge is manipulated.

### Non-associative Knowledge May Influence Association Formation Directly

Assuming that associative learning *does* occur via an associative network of some form, the previous two hypotheses do not necessitate that non-associative cognitive processes have any direct impact on how associations form within that network. Rather, they may affect the information that is fed in to the network and what is done with the output that the network returns. One could posit that cognitive interactions of these forms occur and still assume that associative learning is relatively modular in its operations. However, it is worth considering an alternative hypothesis in which learning within associative networks is directly affected by non-associative factors.

Outcome expectation and prediction error form the centerpiece of many associative learning rules and the obvious and most effective point of interaction of non-associative knowledge with associative processes. Changes to the outcome expectation have profound effects on the updating and thus the structure of the associative network representing the relationships between current cues and outcomes, even if the outcome expectation is more a result of the associative learning system than a part of it.

Since associative learning is often assumed to be proportional to prediction error and predictions can often be made on the basis of both associative and non-associative information, an obvious way in which a direct non-associative influence might occur would be if prediction error was a function of all sources of outcome expectation, and not just associative prediction. In this case, controlled cognitive operations based in metacognition and reasoning could have a significant impact on a key variable that determines trial-to-trial variations in associative learning. Thus variations in cognitive processes could have a lasting impact on the course of associative learning even though association formation lawfully follows a basic learning rule.

**FIGURE 4:**
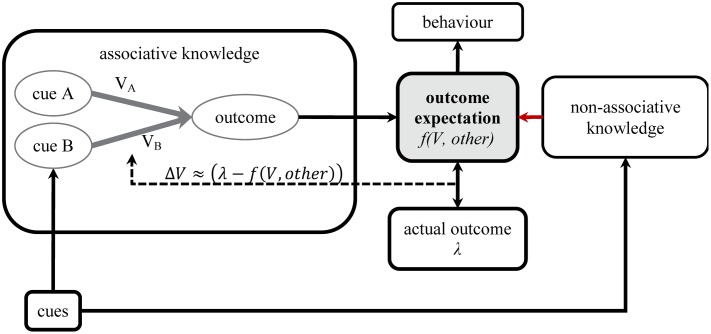
**Non-associative knowledge may influence the outcome expectation that is directly involved in association formation**.

**Figure 4** shows how this interaction of non-associative knowledge with the associative network could work. Crucially, the associative network does not contain additional or enriched representations of information about the cover-story or the outcome and the links are still simple quantitative links that contain no qualitative or structural information about the relationship between the events. It is also important to note that there are no higher-order deductive reasoning processes assumed to be responsible for optimizing and changing the network. Indeed, the associative network in the box in **Figure 4** is exactly the same as that in **Figure 1**, and when a cue is presented, it activates its associative representation and all associatively linked events. The activation of the outcome representation therefore depends on the strength of the associative link and we assume that its retrieval remains a key source of evidence in deciding whether to predict the outcome or not.

However, it is not the only source of evidence as the cues, the learning situation, or the retrieval of the outcome representation itself can trigger other mental processes. After the associative retrieval of the outcome, this knowledge is used to re-evaluate and adjust the expectation of the outcome. The final outcome expectation is then a function of both the strength of the associative links between the presented cues and the outcome and any other information that the learner perceives as being relevant [*f(V, other)*].

One source of non-associative influence is the extent to which the individual reflects upon their own learning and thought processes, that is metacognitive processes. This may be a strong source of variance across different procedures and across individuals and if associative learning is sensitive to the operations of metacognition (in any one of the ways outlined earlier) then this could be a major source of variance in cue competition and other learning phenomena. An obvious way in which metacognition may be relevant to prediction error is the possibility that associative predictions are evaluated and potentially revised by the individual prior to observing the relevant outcome. We describe this re-evaluation as being metacognitive as it relies on assessment of the outcome expectation and some cognizance of the source of that expectation. Thus we typify the process as being very explicit and probably quite variable between individuals and between learning contexts. We will consider an example in relation to blocking.

Associative learning models all assume some degree of generalization between trials that have cues in common. In the case of blocking, pretraining with A+ leads to an expectation of the outcome in the presence of A. This expectation generalizes to AB+ trials. As described above, the default assumption of many associative learning theories is that the associative strengths of the cues that are present will combine in an additive fashion (Rescorla and Wagner, 1972), although there are many hypothesized reasons why this summation might be less than perfectly additive (see McLaren and Mackintosh, 2000, 2002; Wagner and Brandon, 2001; Harris, 2006; Harris and Livesey, 2010; Thorwart et al., 2012 to name just a few). Thus the process that provides a means of generalization is assumed to automatically produce an expectation of the outcome based on some combination of the associative strengths of the cues present. This assumption is based partly on direct evidence of summation in human and animal learning (see for example Myers et al., 2001; Pearce, 2002; Soto et al., 2009; Thorwart et al., 2016) but also on the fact that it is necessary for the associative account of the blocking effect and that the blocking effect is found in diverse and various circumstances and paradigms, indicating that the additivity rule is in fact the default mode by which our learning system operates.

In contrast, when an individual is deliberately engaged in the task of trying to understand the general rules by which relationships between cues and outcomes abide, they may have reason to question this simple summative principle and they may do so to differing to degrees depending on the individual and the context in which they experience the cues and outcomes. We might assume that the process operates according to the following. In phase 1 of a blocking experiment, in addition to forming an association between A and the outcome, the participant has episodic memory of witnessing certain trial types (e.g., A+) and entertains beliefs about the relationship between A and the outcome. In the second stage, the current trial type (AB+) has some overlap with previous experience and associative memory results in retrieval of the outcome representation or increased activation of the outcome representation. This would normally result in an associatively retrieved expectation that the outcome will occur. The learner might accept this expectation at face value and thus will not be particularly surprised to find that the outcome occurs again on this new AB trial. However, the participant may also notice that the current trial type (AB+) is not the same as those previously witnessed. Although the participant has strongly retrieved the outcome representation, they might question whether their expectation of its occurrence is accurate given their uncertainty about the perceived change in trial type. The learner may acknowledge the fact that this is a novel situation, that they don’t know how these indicators operate in combination and entertain the possibility that indicators A and B together might not indicate the same outcome as A alone. The expectation generated on the basis of A+ episodes is consequently moderated, and the learner may regulate their predictions in a way that reduces their expectation of the outcome. That reduction affects both the explicit predictions of the individual and the associative learning that takes place when the participant observes the outcome on that trial. This cautious approach means that the occurrence of the outcome on such trials is still at least partially surprising and its presence should be learned about more effectively. Thus, an associative link between B and the outcome may be established and the blocking effect attenuated.

At face value, this is simply a cognitive description of *external inhibition*, a well-documented effect in animal learning (e.g., Pavlov, 1927) in which the addition of a novel cue reduces the learned response to a previously trained cue. The difference here is that we specifically assume that moderation occurs as a consequence of the participant’s appraisal of what they know about how the cues and outcomes generally operate. Consequently, one can begin to predict different effects on learning in conditions where the cues and outcomes are the same but the causal scenario differs. In a food allergist experiment, a participant might first observe on multiple occasions that their “patient” has consumed Apple and suffers an allergic reaction as a consequence. From this they may form an association between Apple and the reaction, and they may also form a belief that the patient is allergic to Apple. When the patient then eats Apple and Beans in one meal, it seems reasonable to assume that most participants would believe that the patient will suffer an allergic reaction because Apple was eaten. But what of a situation in which the cues are unknown drugs that cause or prevent side effects, or symptoms of a hitherto unknown disease? Is it reasonable to assume that if a patient suffers a migraine after being given Melixil, they will also suffer a migraine when given Melixil and Andrum? Many people might be considerably less sure of this, given that they know nothing about the drugs and have little relevant experience to draw on. One might therefore assume that the expectation of the outcome generated by Melixil (cue A) will be moderated by the uncertainty that the individual feels about the scenario, about the way cues interact, or the reliability of their effects (using this drug scenario, Lee and Livesey, 2012 found no evidence of blocking).

The hypothesis being entertained here is that uncertainty about new trial types may increase the amount that is learned about a redundant cue. The assumption is that factors that increase the uncertainty of a participant about the current learning situation decreases blocking. An extension of this hypothesis would further predict that participants will learn less about the blocking cue B when their natural assumptions about cause and effect in a given scenario are not contradicted by instructions or pretraining. That is, if the participant *feels* well-informed and confident about their understanding of the situation, they may show less evidence of learning about redundant cues.

A hypothesis of this sort is applicable to the influence of non-associative knowledge about the additivity of outcome properties, and specifically how this impacts cue competition. Most causal judgment experiments present deterministic relationships where the probability of the outcome is either 0 or 1 depending on the cue or cues presented, and the presentation of the outcome consists of little more than a label or picture. Therefore the method of presenting the outcome to participants lacks the clarity of information needed to determine whether the outcome is truly additive or non-additive. Lovibond et al. (2003) suggested that this is the reason that blocking is typically fairly weak in human causal learning experiments, because not all participants maintain an assumption of outcome additivity during the experiment. They set about testing the effect of outcome additivity assumptions by giving one group of participants pretraining that explicitly demonstrated the additive nature of the outcome and another group of participants explicit pretraining demonstrating that the outcome was the same magnitude whether there were one or two causes present. The additive group received pretraining in which two cues, which were unconnected to the cues A and B of the actual blocking training, each led to the outcome (X+Y+) and their compound led to an even stronger outcome (XY++, e.g., a stronger allergic response). This group subsequently displayed significantly larger blocking than the non-additive group, which received pre-training in which the compound led to the same outcome as the single cues (X+ Y+ XY+). This result has been replicated in several studies (e.g., Livesey and Boakes, 2004; Beckers et al., 2005a; Mitchell et al., 2005).

This is problematic for associative accounts as no associative knowledge about A and B is established in the pretraining. A common explanation offered is that additivity assumptions encourage deductive reasoning, which results in a conclusion that the blocked cue is not a cause of the outcome (e.g., see Mitchell and Lovibond, 2002; Lovibond et al., 2003; Beckers et al., 2005a). While this explanation is certainly very plausible, additive pretraining like X+ Y+ XY++, which is usually accompanied by very explicit instructions about cue additivity, also removes any uncertainty about the way cues combine in a particular learning situation and in this way could influence the outcome prediction and therefore the prediction error on AB+ trials. As A is known to lead to the outcome, the learner will indeed be unsurprised to find that the outcome occurs again on this new AB trial and no prediction error will occur. However, after non-additive X+Y+ XY+ training, participants still know very little about the way the cues combine. The participant may entertain the hypothesis that the influence of the cues is somehow normalized or that there is a ceiling effect masking the summative effects. If uncertainty at a metacognitive level reduces the outcome expectation, prediction error will increase when AB+ trials are experienced and thus more associative learning takes place when the participant observes the outcome on that trial.

One result that clearly conflicts with this explanation is Beckers et al.’s (2005a) finding that manipulating assumptions about additivity *after* the trial-by-trial learning has already taken place still influences the strength of the blocking effect. It is clearly implausible that the operations of an associative network at the time of learning could be influenced by this later non-associative knowledge. However, non-associative knowledge does not need to change the operations of an associative network at the time of learning but only the impact of the associative knowledge on performance in the test phase after learning, either by influencing the outcome expectation directly or by changing the expression of the associative prediction, as described above. The experience of additional cues between training and test might increase the influence of non-associative knowledge on the outcome expectation by increasing uncertainty – if only for the additional time that has passed between training of the compounds AB+ and CD+ and testing the cues B, C, and D. In this case, blocking under the additive condition may be enhanced because causal ratings for the cues are only weakly related to associative memory and are moderated by the reasoning that additivity instructions strongly encourage.

We have described how an unfamiliar context or unfamiliar cues like unknown drug names will increase the uncertainty of learning situation and how this can explain why it is much harder to find blocking in one scenario than in another. In Waldmann and Holyoak (1992), participants showed less blocking in the diagnostic than in the predictive condition. While the cues were always the same stimuli, participants in their predictive task had to learn whether certain cues would elicit a new kind of emotional response in observers. In contrast, participants in the diagnostic task saw the same features redefined as symptoms of a disease and had to learn which symptoms were diagnostic for the disease. We would argue that the diagnostic learning situation increased uncertainty, for instance because the cover story established that the outcome actually precedes the cues in real life, so that participants were in a situation where they had to “predict” an outcome that had already happened. Furthermore, participants in the diagnostic situation have to take into account alternative diseases as causes of the observed symptoms (Waldmann, 2001). For example, even though fever may be an effect of flu, it has many alternative causes, which participants cannot rule out easily within the learning situation and thus increase the uncertainty about their prediction.

## Issues, Limitations, and Future Directions

The scope of our discussion has been necessarily highly selective and has avoided several issues that are obviously important. As we have noted, we make no attempt here to specify in any way how non-associative knowledge is acquired, and define it simply as cognitive influences that associative networks make no attempt to explain. This undoubtedly belies the complexities involved in acquiring such information. In describing three basic ways how non-associative knowledge might influence learning in an associative learning system, we have also avoided consideration of how their effects might combine. It might well be that sources of non-associative knowledge influence the processing of the cues, the translation of the outcome expectation in behavior as well as the expectation of the outcome directly at the same time. However, for sake of the theoretical exercise, we have left the interaction of all three possible mechanisms out of consideration.

We have chosen to focus our discussion on results from causal and contingency learning paradigms. These results, among others, established the relevance of non-associative knowledge in human causal learning. We would argue that the setting of contingency and causal experiments makes them particularly receptive to such information because they typically rely on explicit and self-paced judgements and since they usually invite the individual to entertain a fictitious scenario in which their previous knowledge may come to bear (even though participants are usually encouraged to ignore what they know about similar causal relationships in the real world). In classical conditioning studies, the experimental situation does not contain much non-associative information that could show an influence on learning. In the extreme case, participants are given no other instruction than to sit in front of a computer screen and pay close attention to it. Far more contextual information is given in human causal learning studies and the experimental situation is thus more likely to encourage activation of non-associative knowledge. However, this does not mean that beliefs and expectations based on non-associative knowledge do not affect classical conditioning and other forms of human learning. At least some studies support the notion that non-associative knowledge affects the learning of conditioned responses as well. For instance, Mitchell and Lovibond (2002) showed that skin conductance conditioning is sensitive to information about outcome additivity given in the verbal instructions. They observed significant effects only when participants received verbal instructions emphasizing the additivity rule whereas blocking was not evident when the instruction introduced a non-additivity rule. Therefore, we assume that these issues are relevant to all forms of human associative learning and extend beyond the limited selection of procedures and phenomena that we have discussed here.

The account we offer here necessarily involves non-associative processes impacting upon observable behavior (i.e., performance) as well as on the formation of associations (i.e., learning). As such, the large number of studies exploring non-associative factors in associative learning – many of which show that instructions, pretraining, and cover stories affect causal and contingency learning – do not offer unique support for, or refutation of, this approach because most can be explained in terms of a performance-level effect alone. To properly test the hypotheses outlined above, a different approach is required, one in which performance-level and learning-level influences can be dissociated. Applying this logic to blocking in causal learning, instructional manipulations are required which can be expected to change participants’ predictions during learning of the AB+ compound *without* resulting in global changes to the way the ratings scale is used at test. There is also still a general need to examine how potential differences in learning manifest differently depending on the properties of the test measure. Although recent work has revealed much about the way blocking is sensitive to causal assumptions, researchers have typically been less concerned with the general properties of the measure itself, even though these properties may strongly affect the potential to observe cue competition effects. The presence of ceiling effects on the strength of ratings provides a simple example of this. As previously noted, using a test measure in which ratings are generally close to ceiling could mask a blocking effect in non-causal scenarios even if the causal scenario made no difference to the strength of learning about competing cues. This simple possibility alone is cause to think seriously about the basic properties of the test measure and is indicative of a more general problem with comparing blocking effects across different conditions. After all, the magnitude of blocking is a *difference* between the judgments made for two types of cue (blocked vs. control), and is often measured on a ratings scale with unknown psychometric properties. Comparing the magnitude of two differences on a measurement scale that is at best ordinal in nature is a risky exercise.

Beyond cue competition, procedures in which associative predictions and non-associative expectation can be directly pitted against each other may be particularly useful for testing the hypotheses outlined in this article. As mentioned above, such examples do exist, though they are relatively rare. Two that might prove useful are Perruchet’s (1985) dissociation between the strength of anticipatory responding and explicit ratings of outcome expectancy and Shanks and Darby’s (1998) dissociation between similarity-based and rule-based generalization.

Perruchet’s dissociation emerged originally in classical human eye-blink conditioning. Perruchet (1985) arranged a partial reinforcement schedule in which the same tone cue played on every trial, but was followed on just 50% of trials by the outcome – an irritant (a puff of air delivered to the eye) that elicits an eyeblink. A conditioning procedure of this kind usually leads to the development of anticipatory eyeblinks during the tone cue in expectation of the airpuff. The randomization of the two trial types (cue-outcome and cue-alone) meant that the trial types sometimes remained the same over several consecutive trials, and sometimes alternated frequently, resulting in short runs of just one or two of the same trial type. When Perruchet arranged the analysis based on the length of the preceding run of trials, he found a pattern of anticipatory eyeblinks that followed the pattern one would expect from conditioning based on basic associative principles. Runs of cue-outcome trials increased anticipatory behavior as a function of the length of the run, whereas runs of cue-alone trials decreased anticipatory behavior as a function of the run length. However, when he asked participants to indicate explicitly how much they expected the airpuff on the next trial, their pattern of expectancies was the opposite; Runs of cue-outcome trials decreased expectancy ratings as a function of run length, whereas runs of cue-alone trials increased expectancy ratings as a function of the run length. This pattern follows a classic gambler’s fallacy effect and is inconsistent with the predictions of associative networks. The result has now been replicated across several paradigms involving classical conditioning and voluntary responding (see Perruchet, 2015 for a review). Current debates about the validity of this dissociation center around whether the pattern observed in anticipatory behavior is a *bona fide* example of associative learning (e.g., Weidemann et al., 2009, 2016; Barrett and Livesey, 2010; Mitchell et al., 2010) and whether participants truly hold these two conflicting belief biases concurrently (Livesey and Costa, 2014; Lee Cheong Lem et al., 2015). However, to date there has been no attempt to explore how these beliefs affect future learning. For instance, after a long run of trials on which the outcome has occurred, if another cue-outcome pairing occurs then the prediction error based on associative mechanisms should be relatively small but prediction error based on explicit expectancy should be relatively high.

The Shanks-Darby patterning task was developed specifically to create opposing influences on generalization within a causal learning task. Shanks and Darby (1998) trained participants to solve multiple examples of a positive patterning (e.g., A-/B-/AB+) and negative patterning (e.g., C+/D+/CD-) in a simple food allergist causal learning procedure. In animal learning, conditional discriminations of this variety, and particularly negative patterning, are relatively difficult to acquire (e.g., Harris et al., 2008), and there is at least some evidence that humans too find negative patterning more difficult to learn than positive patterning (Livesey et al., 2011; Thorwart et al., 2016). Associative networks generally anticipate this difference because the summation of associations formed to the single stimuli in negative patterning (C+ and D+ trials) provides a particularly strong and incorrect prediction for the compound (CD-). However, from an abstract relational perspective, positive and negative patterning possess the same complexity; they are perfect examples for a simple rule that the outcome of the compound is always the opposite of the outcome of the single cues (Shanks and Darby, 1998; Lachnit et al., 2001, 2002; Harris and Livesey, 2008; Cobos et al., 2016). Capitalizing on this simple relational property, Shanks and Darby also trained participants on a series of single cues (I+/J+/M-/N-) and compounds (KL-/OP+), and later tested how participants would predict the consequences of these cues in novel combinations (e.g., IJ?; MN?) or as singles cues (K? L?; O? P?). The authors observed that a subset of participants showed a generalization pattern consistent with this opposites rule such that they predicted the outcome would occur after MN, K, and L and predicted that it would not occur after IJ, O, and P. This pattern of behavior is hard to reconcile with an associative network which derives its predictions based on feature overlap and thus would predict the exact opposite pattern. Even if knowledge gained about the complete patterning discriminations (A- B- AB+; C+ D+ CD-) is represented within the associative system, it would not be activated in the IJ? or MN? test trials and thus influence the outcome expectation. Maes et al. (2015) have shown that this pattern of abstract rule generalization is absent from the behavior of rats and pigeons, which appear to generalize mainly in ways consistent with associative learning principles. Cobos et al. (2016) showed the same is true for humans when using a cued-response priming task, whereas verbal ratings were consistent with rule-based generalization. Furthermore, the use of rule-based generalization has been shown to be related to working memory, cognitive reflection, and strategic model-based choice in other instrumental learning tasks (Wills et al., 2011a,b; Don et al., 2015, 2016). However, as with the Perruchet effect, researchers have not yet explored whether these competing forms of generalization have an impact on the strength of future learning. Given that several cognitive correlates of rule extraction can be used to predict which individuals are most likely to use a relational rule in this task, predictions can be made about which individuals should find it surprising when a new trial type violates the rule and which should not.

These avenues for future research are among several that might be fruitful for testing how associative predictions and expectations based on non-associative factors might contribute to new learning. Given that most of the current evidence is consistent with multiple theoretical accounts (including those that retain and those that reject classical association formation as a key explanatory construct), devising new experimental designs is essential for the advancement of the field.

## Conclusion

Having valid and reliable expectations about future events is one of the most essential and necessary conditions for the adaptivity of human behavior. Associative learning theories have offered a very successful account of how humans obtain these expectations and how they update and optimize them whenever these expectations are violated. However, by necessity, formal implementations of these theories in associative networks have a limited scope, which does not capture the influence of a variety of other cognitive factors on our learned judgments and expectations. We have explored three ways how these sources of non-associative knowledge can affect associative learning without changing the fundamental principles of such an associative learning system. We argue that recent theorists have failed to give these possibilities due credence and, even though there is no specific evidence for any of them, they offer plausible ways in which an associative learning and memory system may contribute to judgments and expectations that is consistent with most of the available evidence. Future research is needed to examine whether and how associative predictions and other sources of expectations contribute to future associative learning.

## Author Contributions

AT and EL were equally responsible for the conception, drafting, and revising of the paper.

## Conflict of Interest Statement

The authors declare that the research was conducted in the absence of any commercial or financial relationships that could be construed as a potential conflict of interest.

## Footnotes

1 There are successful attempts to capture at least some of the effects we refer to as non-associative within a purely associative learning framework. For instance, Haselgrove (2010) has successfully modeled the effects of some forms of pretraining on blocking by appealing to the role played by common elements. However, these attempts rely on assumptions that should not necessarily be easily generalized to other learning designs and paradigms. For instance, a necessary assumption of Haselgrove’s (2010) explanation is strong generalization from previously trained cues to the critical test cues and is most clearly applicable to designs in which most or all stimuli are paired with the outcome. In contrast allergist tasks are usually run with multiple filler cues that do not predict the outcome and most participants in these experiments show very clear discrimination between trained and novel cues.

## References

[B1] Annau Z. Kamin L. J. (1961). The conditioned emotional response as a function of intensity of the US. *J. Comp. Physiol. Psychol.* 54 428–432. 10.1037/h0042199 13683658

[B2] Barrett L. C. Livesey E. J. (2010). Dissociations between expectancy and performance in simple and two-choice reaction-time tasks: a test of associative and nonassociative explanations. *J. Exp. Psychol. Learn. Mem. Cogn.* 36 864–877. 10.1037/a0019403 20565206

[B3] Beckers T. De Houwer J. Pineno O. Miller R. R. (2005a). Outcome additivity and outcome maximality influence cue competition in human causal learning. *J. Exp. Psychol. Learn. Mem. Cogn.* 31 238–249. 15755242 10.1037/0278-7393.31.2.238

[B4] Beckers T. Van den Broeck U. V. Renne M. Vandorpe S. Houwer J. D. Eelen P. (2005b). Blocking is sensitive to causal structure in 4-year-old and 8-year-old children. *Exp. Psychol.* 52 264–271. 10.1027/1618-3169.52.4.264 16302535

[B5] Blanco F. Baeyens F. Beckers T. (2014). Blocking in human causal learning is affected by outcome assumptions manipulated through causal structure. *Learn. Behav.* 42 185–199. 10.3758/s13420-014-0137-y 24737045

[B6] Blanco F. Matute H. Vadillo M. A. (2012). Mediating role of activity level in the depressive realism effect. *PLoS ONE* 7:e46203 10.1371/journal.pone.0046203 PMC3459889 23029435

[B7] Boddez Y. Haesen K. Baeyens F. Beckers T. (2014). Selectivity in associative learning: a cognitive stage framework for blocking and cue competition phenomena. *Front. Psychol.* 5:1305 10.3389/fpsyg.2014.01305 PMC4228836 25429280

[B8] Cobos P. L. Gutiérrez-Cobo M. J. Morís J. Luque D. (2016). Dependent measure and time constraints modulate the competition between conflicting feature-based and rule-based generalization processes. *J. Exp. Psychol. Learn. Mem. Cogn.* 10.1037/xlm0000335 [Epub ahead of print]. 27841447

[B9] Colagiuri B. Livesey E. J. (2016). Contextual cuing as a form of nonconscious learning: theoretical and empirical analysis in large and very large samples. *Psychonomic Bull. Rev.* 23 1996–2009. 10.3758/s13423-016-1063-0 27220995

[B10] De Houwer J. (2009). The propositional approach to associative learning as an alternative for association formation models. *Learn. Behav.* 37 1–20. 10.3758/LB.37.1.1 19122048

[B11] De Houwer J. Beckers T. Glautier S. (2002). Outcome and cue properties modulate blocking. *Q. J. Exp. Psychol.* 55A, 965–985. 10.1080/02724980143000578 12188522

[B12] Dickinson A. Shanks D. Evenden J. (1984). Judgement of act-outcome contingency: the role of selective attribution. *Q. J. Exp. Psychol.* 36 29–50. 10.1080/14640748408401502

[B13] Don H. J. Goldwater M. B. Otto R. Livesey E. J. (2016). Rule abstraction, model-based choice and cognitive reflection. *Psychonomic Bull. Rev.* 23 1615–1623. 10.3758/s13423-016-1012-y 26907600

[B14] Don H. J. Goldwater M. B. Otto R. A. Livesey E. J. (2015). “Connecting rule-abstraction and model-based choice across disparate learning tasks,” in *Proceedings of the 37th Annual Conference of the Cognitive Science Society*, eds Noelle D. Dale R. Warlaumont A. Yoshimi J. Matlock T. Jennings C. (Pasadena, CA: Cognitive Science Society), 590–595.

[B15] Don H. J. Livesey E. J. (2015). Resistance to instructed reversal of the learned predictiveness effect. *Q. J. Exp. Psychol.* 68 1327–1347. 10.1080/17470218.2014.979212 25383751

[B16] Fiedler K. Juslin P. (2006). *Information Sampling and Adaptive Cognition.* New York, NY: Cambridge University Press.

[B17] Goujon A. Didierjean A. Thorpe S. (2015). Investigating implicit statistical learning mechanisms through contextual cueing. *Trends Cogn. Sci.* 19 524–533. 10.1016/j.tics.2015.07.009 26255970

[B18] Gredebäck G. Winman A. Juslin P. (2000). “Rational assessments of covariation and causality,” in *Proceedings of the 22nd Annual Conference of the Cognitive Science Society*, eds Gleitman L. R. Joshi K. (Mahwah, NJ: Erlbaum), 190–195.

[B19] Harris J. Livesey E. Gharaei S. Westbrook F. (2008). Negative patterning is easier than a biconditional discrimination. *J. Exp. Psychol. Anim. Behav. Process.* 34 494–500. 10.1037/0097-7403.34.4.494 18954233

[B20] Harris J. A. (2006). Elemental representations of stimuli in associative learning. *Psychol. Rev.* 113 584–605. 10.1037/0033-295X.113.3.584 16802882

[B21] Harris J. A. Livesey E. J. (2008). Comparing patterning and biconditional discriminations in humans. *J. Exp. Psychol. Anim. Behav. Process.* 34 144–154. 10.1037/0097-7403.34.1.144 18248121

[B22] Harris J. A. Livesey E. J. (2010). An attention-modulated associative network. *Learn. Behav.* 38 1–26. 10.3758/LB.38.1.1 20065345

[B23] Haselgrove M. (2010). Reasoning rats or associative animals? A common-element analysis of the effects of additive and subadditive pretraining on blocking. *J. Exp. Psychol. Anim. Behav. Process.* 36 296–306. 20384408 10.1037/a0016603

[B24] Kamin L. J. (1968). “Attention-like processes in classical conditioning,” in *Miami Symposium on the Prediction of Behavior: Aversive Stimulation*, ed. Jones M. R. (Miami, FL: University of Miami Press), 9–33.

[B25] Lachnit H. Kinder A. Reinhard G. (2002). Are rules applied in Pavlovian electrodermal conditioning with humans general or outcome specific? *Psychophysiology* 39 380–387. 10.1017/S0048577201393125 12212657

[B26] Lachnit H. Lober K. Reinhard G. Kinder A. (2001). Evidence for the application of rules in Pavlovian electrodermal conditioning with humans. *Biol. Psychol.* 56 151–166. 10.1016/S0301-0511(01)00067-9 11334701

[B27] Lashley K. S. Wade M. (1946). The Pavlovian theory of generalization. *Psychol. Rev.* 53 72–87. 10.1037/h0059999 21023320

[B28] Le Pelley M. E. Mitchell C. J. Beesley T. George D. N. Wills A. J. (2016). Attention and associative learning in humans: an integrative review. *Psychol. Bull.* 142 1111–1140. 10.1037/bul0000064 27504933

[B29] Lee J. C. Livesey E. J. (2012). Second-order conditioning and conditioned inhibition: influences of speed versus accuracy on human causal learning. *PLoS ONE* 7:e49899 10.1371/journal.pone.0049899 PMC3509133 23209613

[B30] Lee Cheong Lem V. A. Harris J. A. Livesey E. J. (2015). Testing the limits of the Perruchet effect in choice response time tasks. *J. Exp. Psychol. Anim. Learn. Cogn.* 41 385–394. 10.1037/xan0000079 26301613

[B31] Livesey E. Lee J. Shone L. (2013). “The relationship between blocking and inference in causal learning,” in *Proceedings of the 35th Annual Meeting of the Cognitive Science Society (COGSCI 2013)*, eds Knauff M. Pauen M. Sebanz N. Wachsmuth I. (Austin, TX: Cognitive Science Society).

[B32] Livesey E. J. Boakes R. A. (2004). Outcome additivity, elemental processing and blocking in human causality judgements. *Q. J. Exp. Psychol.* 57B, 361–379. 10.1080/02724990444000005 15513261

[B33] Livesey E. J. Costa D. S. J. (2014). Automaticity and conscious control in single and choice response time versions of the Perruchet effect. *Q. J. Exp. Psychol.* 67 646–664. 10.1080/17470218.2013.824014 23972053

[B34] Livesey E. J. Harris J. A. (2008). What are flexible representations? commentary on Melchers, Shanks and Lachnit. *Behav. Process.* 77 437–439. 10.1016/j.beproc.2007.09.006 17988806

[B35] Livesey E. J. Harris J. A. (2009). Is there room for simple links in a propositional mind? (Commentary on Mitchell etal.). *Behav. Brain Sci.* 32 212–213. 10.1017/S0140525X09001010

[B36] Livesey E. J. Thorwart A. Harris J. A. (2011). Comparing positive and negative patterning in human learning. *Q. J. Exp. Psychol.* 64 2316–2333. 10.1080/17470218.2011.605153 22026453

[B37] Lovibond P. F. Been S. L. Mitchell C. J. Bouton M. E. Frohardt R. (2003). Forward and backward blocking of causal judgment is enhanced by additivity of effect magnitude. *Mem. Cogn.* 31 133–142. 10.3758/BF03196088 12699149

[B38] Luque D. Cobos P. L. López F. J. (2008). Interference between cues requires a causal scenario: favorable evidence for causal reasoning models in learning processes. *Learn. Mot.* 39 196–208. 10.1016/j.lmot.2007.10.001

[B39] Luque D. Flores A. Vadillo M. A. (2013). Revisiting the role of within-compound associations in cue-interaction phenomena. *Learn. behav.* 41 61–76. 10.3758/s13420-012-0085-3 22753000

[B40] Mackintosh N. J. (1975). A theory of attention: variations in the associability of stimuli with reinforcement. *Psychol. Rev.* 82 276–298. 10.1037/h0076778

[B41] Mackintosh N. J. (1976). Overshadowing and stimulus intensity. *Anim. Learn. Behav.* 4 186–192. 10.3758/BF03214033 964444

[B42] Maes E. Filippo G. D. Inkster A. B. Lea S. E. G. Houwer J. D. D’Hooge R. (2015). Feature- versus rule-based generalization in rats, pigeons and humans. *Anim. Cogn.* 18 1267–1284. 10.1007/s10071-015-0895-8 26188712 PMC4607717

[B43] Matute H. (1996). Illusion of control: detecting response-outcome independence in analytic but not in naturalistic conditions. *Psychol. Sci.* 7 289–293. 10.1111/j.1467-9280.1996.tb00376.x

[B44] Matute H. Arcediano F. Miller R. R. (1996). Test question modulates cue competition between causes and between effects. *J. Exp. Psychol. Learn. Mem. Cogn.* 22 182–196. 10.1037/0278-7393.22.1.182 8648285

[B45] McLaren I. P. L. Mackintosh N. J. (2000). An elemental model of associative learning: I. Latent inhibition and perceptual learning. *Anim. Learn. Behav.* 28 211–246. 10.3758/BF03200258

[B46] McLaren I. P. L. Mackintosh N. J. (2002). Associative learning and elemental representation: II. Generalization and discrimination. *Anim. Learn. Behav.* 30 177–200. 10.3758/BF03192828 12391785

[B47] Melchers K. G. Shanks D. R. Lachnit H. (2008). Stimulus coding in human associative learning: flexible representations of parts and wholes. *Behav. Process.* 77 413–427. 10.1016/j.beproc.2007.09.013 18031954

[B48] Mitchell C. J. De Houwer J. Lovibond P. F. (2009). The propositional nature of human associative learning. *Behav. Brain Sci.* 32 183–198. 10.1017/S0140525X09000855 19386174

[B49] Mitchell C. J. Griffiths O. Seeto J. Lovibond P. F. (2012). Attentional mechanisms in learned predictiveness. *J. Exp. Psychol. Anim. Behav. Process.* 38 191–202. 10.1037/a0027385 22369199

[B50] Mitchell C. J. Lovibond P. F. (2002). Backward and forward blocking in human electrodermal conditioning: blocking requires an assumption of outcome additivity. *Q. J. Exp. Psychol.* 55B, 311–329. 10.1080/02724990244000025 12350284

[B51] Mitchell C. J. Lovibond P. F. Condoleon M. (2005). Evidence for deductive reasoning in blocking of causal judgments. *Learn. Mot.* 36 77–87. 10.1016/j.lmot.2004.09.001

[B52] Mitchell C. J. Wardle S. G. Lovibond P. F. Weidemann G. Chang B. P. I. (2010). Do reaction times in the Perruchet effect reflect variations in the strength of an associative link? *J. Exp. Psychol. Learn. Mem. Cogn.* 36 567–572. 10.1037/a0018433 20192552

[B53] Morís J. Cobos P. L. Luque D. López F. J. (2014). Associative repetition priming as a measure of human contingency learning: evidence of forward and backward blocking. *J. Exp. Psychol. Gen.* 143 77–93. 10.1037/a0030919 23230993

[B54] Myers K. Vogel E. Shin J. Wagner A. (2001). A comparison of the Rescorla-Wagner and Pearce models in a negative patterning and a summation problem. *Anim. Learn. Behav.* 29 36–45. 10.3758/BF03192814

[B55] Pavlov I. P. (1927). *Conditioned Reflexes: An Investigation of the Physiological Activity of the Cerebral Cortex*. Oxford: Oxford University Press. 10.5214/ans.0972-7531.1017309 PMC4116985 25205891

[B56] Pearce J. M. (2002). Evaluation and development of a connectionist theory of configural learning. *Anim. Learn. Behav.* 30 73–95. 10.3758/BF03192911 12141138

[B57] Pearce J. M. Hall G. (1980). A model for Pavlovian learning: variations in the effectiveness of conditioned but not of unconditioned stimuli. *Psychol. Rev.* 87 532–552. 10.1037/0033-295X.87.6.532 7443916

[B58] Perruchet P. (1985). A pitfall for the expectancy theory of human eyelid conditioning. *Pavlov. J. Biol. Sci.* 20 163–170. 4069791 10.1007/BF03003653

[B59] Perruchet P. (2015). Dissociating conscious expectancies from automatic link formation in associative learning: a review on the so-called Perruchet effect. *J. Exp. Psychol. Anim. Learn. Cogn.* 41 105–127. 10.1037/xan0000060 25867141

[B60] Redhead E. S. Pearce J. M. (1995). Stimulus salience and negative patterning. *Q. J. Exp. Psychol.* 48 67–83. 7740125

[B61] Rescorla R. A. Wagner A. R. (1972). “A theory of Pavlovian conditioning: Variations in the effectiveness of reinforcement and nonreinforcement,” in *Classical Conditioning II: Current Research and Theory*, eds Black A. H. Prokasy W. F. (New York, NY: Appleton Century Crofts), 64–99. 10.1037/a0030892

[B62] Shanks D. R. (2007). Associationism and cognition: human contingency learning at 25. *Q. J. Exp. Psychol.* 60 291–309. 10.1080/17470210601000581 17366302

[B63] Shanks D. R. Darby R. J. (1998). Feature- and rule-based generalization in human associative learning. *J. Exp. Psychol. Anim. Behav. Process.* 24 405–415. 10.1037//0097-7403.24.2.136 9556907

[B64] Shanks D. R. Lopez F. (1996). Causal order does not affect cue selection in human associative learning. *Mem. Cogn.* 24 511–522. 10.3758/BF03200939 8757499

[B65] Shone L. T. Harris I. M. Livesey E. J. (2015). Automaticity and cognitive control in the learned predictiveness effect. *J. Exp. Psychol. Anim. Learn. Cogn.* 41 18–31. 10.1037/xan0000047 25706543

[B66] Soto F. A. Vogel E. H. Castillo R. D. Wagner A. R. (2009). Generality of the summation effect in human causal learning. *Q. J. Exp. Psychol.* 62 877–889. 10.1080/17470210802373688 19048450

[B67] Steinberg E. E. Keiflin R. Boivin J. R. Witten I. B. Deisseroth K. Janak P. H. (2013). A causal link between prediction errors, dopamine neurons and learning. *Nat. Neurosci.* 16 966–973. 10.1038/nn.3413 23708143 PMC3705924

[B68] Sternberg D. A. McClelland J. L. (2012). Two mechanisms of human contingency learning. *Psychol. Sci.* 23 59–68. 10.1177/0956797611429577 22198929

[B69] Sutherland N. S. Mackintosh N. J. (1971). *Mechanisms of Animal Discrimination Learning.* New York, NY: Academic Press.

[B70] Thorwart A. Lachnit H. (2009). Symmetrical generalization decrements: configural stimulus processing in human contingency learning. *Learn. Behav.* 37 107–115. 10.3758/LB.37.1.107 19122057

[B71] Thorwart A. Lachnit H. (2010). Generalization decrements: further support for flexibility in stimulus processing. *Learn. Behav.* 38 367–373. 10.3758/LB.38.4.367 21048227

[B72] Thorwart A. Livesey E. J. Harris J. A. (2012). Normalization between stimulus elements in a model of Pavlovian conditioning: showjumping on an elemental horse. *Learn. Behav.* 40 334–346. 10.3758/s13420-012-0073-7 22927005

[B73] Thorwart A. Uengoer M. Livesey E. J. Harris J. A. (2016). Summation effects in human learning: evidence from patterning discriminations in goal-tracking. *Q. J. Exp. Psychol.* 10.1080/17470218.2016.1184290 [Epub ahead of print]. 27126385

[B74] Vadillo M. A. Konstantinidis E. Shanks D. R. (2016). Underpowered samples, false negatives, and unconscious learning. *Psychonomic Bull. Rev.* 23 87–102. 10.3758/s13423-015-0892-6 PMC4742512 26122896

[B75] Vadillo M. A. Matute H. M. (2007). Predictions and causal estimations are not supported by the same associative structure. *Q. J. Exp. Psychol.* 60 433–447. 10.1080/17470210601002520 17366310

[B76] Vadillo M. A. Miller R. R. Matute H. M. (2005). Causal and predictive-value judgments, but not predictions, are based on cue–outcome contingency. *Learn. Behav.* 33 172–183. 10.3758/BF03196061 16075837

[B77] Wagner A. R. Brandon S. E. (2001). “A componential theory of Pavlovian conditioning,” in *Handbook of Contemporary Learning Theories*, eds Mowrer R. R. Klien S. B. (Mahwah, NJ: Erlbaum), 23–64.

[B78] Waldmann M. R. (2000). Competition among causes but not effects in predictive and diagnostic learning. *J. Exp. Psychol. Learn. Mem. Cogn.* 26 53–76. 10.1037/0278-7393.26.1.53 10682290

[B79] Waldmann M. R. (2001). Predictive versus diagnostic causal learning: evidence from an overshadowing paradigm. *Psychonomic Bull. Rev.* 8 600–608. 10.3758/BF03196196 11700912

[B80] Waldmann M. R. Holyoak K. J. (1992). Predictive and diagnostic learning within causal models: asymmetries in cue competition. *J. Exp. Psychol. Gen.* 121 222–236. 10.1037/0096-3445.121.2.222 1534834

[B81] Weidemann G. McAndrew A. Livesey E. J. McLaren I. P. L. (2016). Evidence for multiple processes contributing to the Perruchet effect: Response priming and associative learning. *J. Exp. Psychol. Anim. Learn. Cogn.* 42 366–379. 27732048 10.1037/xan0000117

[B82] Weidemann G. Tangen J. M. Lovibond P. F. Mitchell C. J. (2009). Is Perruchet’s dissociation between eyeblink conditioned responding and outcome expectancy evidence for two learning systems? *J. Exp. Psychol. Anim. Behav. Process.* 35 169–176. 10.1037/a0013294 19364226

[B83] Wills A. J. Barrasin T. J. McLaren I. P. L. (2011a). “Working memory capacity and generalization in predictive learning,” in *Proceedings of the 33rd Annual Conference of the Cognitive Science Society*, eds Carlson L. Hölscher C. Shipley T. (Austin, TX: Cognitive Science Society), 3205–3210.

[B84] Wills A. J. Graham S. Koh Z. McLaren I. P. L. Rolland M. D. (2011b). Effects of concurrent load on feature- and rule-based generalization in human contingency learning. *J. Exp. Psychol. Anim. Behav. Process.* 37 308–316. 10.1037/a0023120 21500927

[B85] Yarritu I. Matute H. Vadillo M. A. (2014). Illusion of control: the role of personal involvement. *Exp. Psychol.* 61 38–47. 10.1027/1618-3169/a000225 23948387 PMC4013923

